# A coronary coronary-to-coronary fistula around the main pulmonary artery

**DOI:** 10.1007/s10554-024-03269-7

**Published:** 2024-10-22

**Authors:** Stefan Gherca, Gregor Leibundgut, Jokin Zubizarreta-Oteiza, Philip Haaf

**Affiliations:** 1 Department of Radiology, Clinic of Radiology and Nuclear Medicine, University Hospital Basel, University of Basel, Basel, Switzerland; 2 Department of Cardiology, Cardiovascular Research Institute Basel, University Hospital Basel and University of Basel, Petersgraben 4, Basel, CH-4031 Switzerland; 3 Department of Biomedical Engineering, Medical Additive Manufacturing Research Group (Swiss MAM), University of Basel, Allschwil, Switzerland; 4 Oral and Cranio-Maxillofacial Surgery, University Hospital Basel, Basel, Switzerland

**Keywords:** Coronary fistula, Computed tomography, Invasive coronary angiography, Steal phenomenon, 3D-print, Rubidium position emisssion tomography

## Abstract

Coronary-to-coronary fistulas are rare anomalies, often detected incidentally. These fistulas may involve complex vascular connections around major cardiac structures, such as the pulmonary artery. Functional assessments help determine clinical significance, with management typically involving observation for asymptomatic patients, while symptomatic cases may require intervention to prevent complications.

**Supplementary Information:**

The online version contains supplementary material available at 10.1007/s10554-024-03269-7.

A 63-year-old female with recurrent, atypical chest pain underwent coronary computed tomography angiography (CCTA), which ruled out obstructive coronary artery disease (CAD). CCTA revealed a complex coronary-to-coronary fistula encircling the main pulmonary artery (MPA), fed by a conus artery from the right coronary artery (RCA) and a branch from the proximal left anterior descending artery (LAD). A coronary aneurysm (15 × 15 mm) of the LAD branch suggested a direct communication between RCA and LAD with drainage into the MPA (Fig. 1A-D; Supplement Video S1).

Invasive coronary angiography confirmed the ring-like coronary-to-coronary fistula draining into the MPA (Fig. 1E, F; Supplement Video S2, S3). Fractional flow reserve (FFR) revealed a steal phenomenon in the distal RCA (FFR = 0.74) but not in the distal LAD (FFR = 0.90). Rubidium Positron Emission Tomography showed no scar or ischemia and normal myocardial blood flow reserve (Supplemental Figure 1).

Based on these findings, the patient’s age, and her preferences, a conservative approach was chosen.

Coronary fistulas, often resulting from embryological defects, typically drain into low-pressure vasculature [1]. The widespread use of CCTA has led to more frequent detection of these fistulas [2]. Haemodynamically significant fistulae causing symptoms should be treated interventionally or surgically [3], while asymptomatic patients with small fistulas are usually managed conservatively [1].

**Fig. 1:**
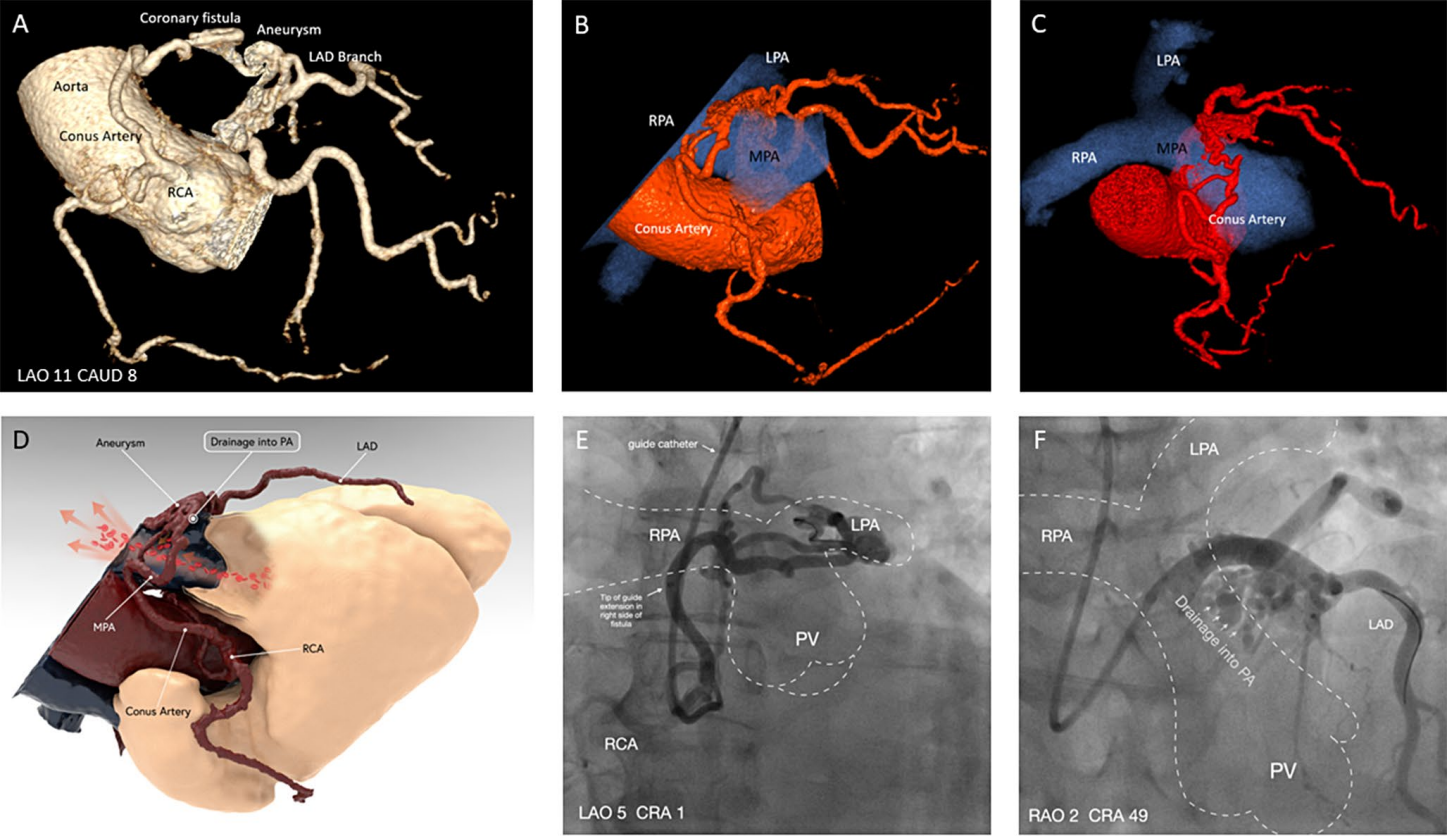
Panel **(A)** Coronary computed tomography angiography 3D volume rendering of the coronary-to-coronary fistula forming a complete circle around the main pulmonary artery (MPA not displayed). **(B**, **C)** Computed tomography 3D model showing the anatomy of the coronary-to-coronary fistula and its relationship with the pulmonary arteries. **(D)** Computed tomography 3D volume rendering of the fistula in relation to MPA. **(E**, ** F)** Invasive coronary angiography in different angulations with sketch of course of the pulmonary arteries and site of drainage of the coronary fistula into the main pulmonary artery

## Electronic supplementary material

Below is the link to the electronic supplementary material.

Supplementary Material 1

Supplementary Material 2

Supplementary Material 3

Supplementary Material 4

## Data Availability

No datasets were generated or analysed during the current study.

## References

[1] Achim A et al (2023) Coronary steal: how many thieves are out there? Eur Heart J 44(30):2805–2814 37264699 10.1093/eurheartj/ehad327

[2] Ouchi K, Sakuma T, Ojiri H (2020) Coronary artery fistula in adults: incidence and appearance on cardiac computed tomography and comparison of detectability and hemodynamic effects with those on transthoracic echocardiography. J Cardiol 76(6):593–600 32636129 10.1016/j.jjcc.2020.06.005

[3] Warnes CA et al (2008) ACC/AHA 2008 guidelines for the management of adults with congenital heart disease. Circulation 118(23):e714–e833 18997169 10.1161/CIRCULATIONAHA.108.190690

