# Supplementary material for: A coronary coronary-to-coronary fistula around the main pulmonary artery
Source: Int J Cardiovasc Imaging. 2024 Oct 22;41(3):639–40. doi: 10.1007/s10554-024-03269-7 (PMC11880091; doi:10.1007/s10554-024-03269-7)
Supplement: Supplementary file 4 — Supplementary Material 4 [file 10554_2024_3269_MOESM4_ESM.docx]

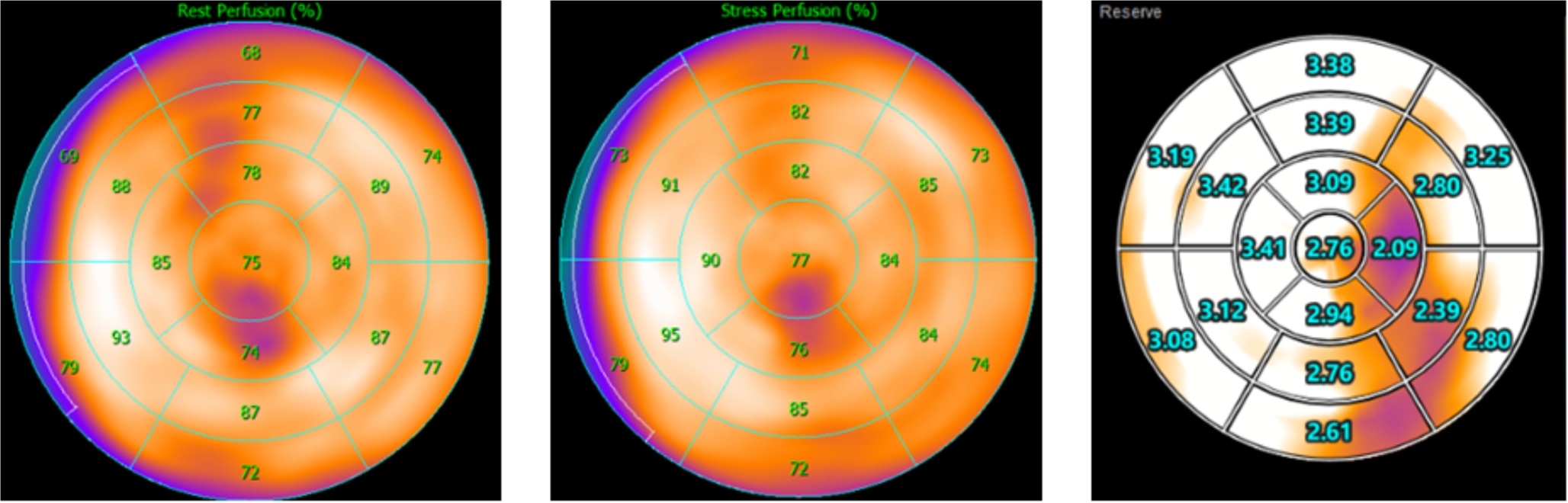


**Supplemental Figure.** Cardiac Rubidium Positron Emission Tomography with Computed Tomography showed no scar, no ischaemia, and normal coronary flow reserve.

**Supplement Video S1**

CT stack with axial Maximum Intensity Projection (MIP) images from caudal to cranial demonstrating the origin of the communicating arteries from the right and left coronary arteries and their proximity to the main pulmonary artery (MPA). The aneurysm of the left coronary branch can be observed in the most cranial images.

**Supplement Video S2 and S3**

Invasive coronary angiography reveals the ring-shaped coronary-to-coronary fistula around the MPA. After cannulation of the conus artery (Supplement Video S2) and the left coronary artery (Supplement Video S3), the MPA demonstrates contrast inflow, confirming drainage from the coronary fistula.
